# Boosting open-label placebo effects in acute induced pain in healthy adults (BOLPAP-study): study protocol of a randomized controlled trial

**DOI:** 10.3389/fmed.2024.1238878

**Published:** 2024-02-14

**Authors:** Matthijs de Leeuw, Mirjam Laager, Jens Gaab, Wilhelm Ruppen, Tobias Schneider

**Affiliations:** ^1^Pain Unit, Clinic for Anesthesia, Intermediate Care, Prehospital Emergency Medicine and Pain Therapy, University Hospital Basel, Basel, Switzerland; ^2^Department of Clinical Research, University of Basel, Basel, Switzerland; ^3^Division of Clinical Psychology and Psychotherapy, Faculty of Psychology, University of Basel, Basel, Switzerland

**Keywords:** open-label placebo, booster, placebo analgesia, acute pain, electrically-evoked pain, hyperalgesia, allodynia

## Abstract

**Introduction:**

Pain is a highly prevalent symptom in the hospital setting, but treatment options remain limited. Harnessing the placebo effect in an ethical manner could provide a new possibility to reduce pain in clinical practice. So called open-label placebos (OLP) have been shown to elicit significant effects in reducing acute pain. But, before implementation, more knowledge concerning the properties of OLPs is needed. This study aims to assess the duration of analgesic effects from OLP and to determine the possibility of boosting such effects.

**Methods and analysis:**

This is the protocol of an ongoing (first patient enrolled in March 2023) single-site randomized trial investigating OLPs in two parts (i.e., substudies). In both parts, pain will be induced in healthy adults using an intradermal electrical stimulation model. Participants in Part 1 will have two study visits: An interventional visit with one OLP injection accompanied by an evidence-based treatment rationale and a control visit with no treatment. For Part 2, participants will be randomized into three groups: (1) A fixed-time “Booster” group including one single repetition of the OLP injection at a fixed time point, (2) an on-demand “Booster” group including one single repetition of the OLP injection on-demand, and (3) a control group who will receive just one OLP injection. Differences in pain ratings over time (using the Numeric Rating Scale) will be analyzed with several two-sample *t*-tests. The time point for a fixed-time “Booster” in Part 2 will be derived from Part 1 with additional statistical tools such as a broken-stick mixed-effect model.

**Discussion:**

This study aims to further characterize the analgesic effects of OLPs. In doing so, it will provide valuable information needed for later implementation of OLPs in clinical practice, where they could play a role in multimodal analgesic concepts.

**Ethics and dissemination:**

The “Ethikkommission Nordwest- und Zentralschweiz” (BASEC 2023-00296) approved the study protocol. Results of the analysis will be submitted for publication in a peer-reviewed journal.

**Clinical Trial Registration:**

This study is registered at ClinicalTrials.gov (NCT05819476) and is listed in the Swiss National Registry at kofam.ch (SNCTP000005470).

## 1 Introduction

Pain is highly prevalent in hospital settings, with up to 84% of adult patients reporting it as a symptom and up to 36% describing it as severe (1). Standard systemic treatment for acute pain mainly consists of multimodal analgesic concepts including acetaminophen, metamizol, non-steroidal anti-inflammatory drugs, and opioids (2–7). However, the use of these drugs is often restricted due to contraindications, side-effects, and adverse events (2–8). Therefore, broadly applicable new treatment options with fewer side-effects would be valuable.

The word placebo is used nowadays to describe sham treatments and “inert” substances like sugar pills and saline injections (9–12). Placebos have been proven to elicit clinically significant effects in various conditions, including pain (9–16). Moreover, due to the “inert” nature of placebos, there are few to no contraindications. Side-effects occurring after the administration of placebos, termed the nocebo effect, are rare and usually stem from the expectation of adverse events (11, 17). So, one could argue that placebos themselves are very safe and could be used in most patients. Nevertheless, ethical concerns about the deceptive nature of placebos (18–20) have prevented their implementation in clinical practice.

To address this issue, it might be possible to prescribe placebos openly, without deception. This concept was proposed and found to be effective back in 1965 (21). Over the last two decades, this topic has become an emerging field of research with several studies focusing on these so-called open-label placebos (OLPs) (22–55). Two meta-analyses summarize clinical OLP studies and highlight significant effects in various conditions, such as allergic rhinitis, menopausal hot flushes, attention deficit hyperactivity disorder, cancer-related fatigue, irritable bowel syndrome, and chronic low back pain (56, 57). Findings on acute pain, though limited, are promising (25–29, 32, 35, 49, 55). To our knowledge, the effects of OLPs on acute pain-associated phenomena, hyperalgesia and allodynia, have only been investigated in one study thus far (55).

As mentioned by the Initiative on Methods, Measurement, and Pain Assessment in Clinical Trials (IMMPACT), characterizing the efficacy of a new analgesic is crucial to its development (58). Attributes of efficacy include onset of effect, maximum analgesic effect, duration of analgesia, and overall analgesic effects across the therapeutic dose range of the drug (58). In a proof-of-concept study from our own research group, we were able to show that OLP injections were able to significantly reduce pain (21% reduction), hyperalgesia and allodynia (47% reduction for both) in a population of healthy adult males experiencing induced pain (55). Although onset of OLP analgesia was nearly immediate, duration could not be observed in full as the analgesic effects lasted beyond the entire 70-min measurement period (55). In a study investigating OLP analgesia in migraine attacks, significant reduction of pain could still be measured two hours after intake of OLP pills (49). To our knowledge, observations over the full duration of OLP analgesia and placebo analgesia are absent. Therapeutic dose range of OLPs – although not for acute pain, but for participant well-being – was investigated by El Brihi et al. (31), but they did not find an influence of dose on their outcomes.

Furthermore, IMMPACT emphasizes the need to establish profiles of new analgesic therapies (58) and recommends the use of both single-dose and multiple-dose study designs (58). In using deceptive placebos, multiple placebos and more frequent intake have been shown to increase effects (59, 60). However, there is – to the best of our knowledge – only one study investigating possible superiority of repeated OLP administration over one-time OLP administration (31), showing no statistically significant influence of OLP repetition on participant well-being. Nevertheless, there are several studies administering multiple-dose OLPs in multiple day interventions on various conditions (e.g., 31, 33–39, 42, 43, 45, 46, 48, 51). But, none of these studies investigated whether repeated OLP intake actually is superior to single-dose intake and none did determine the optimal timing of repeat dosing. Likely, the decision to use multiple-dose OLPs (i.e., two OLP pills twice a day in most studies) comes from weighting the evidence on deceptive placebos mentioned above with evidence suggesting that “simple dosing regimens encourage higher adherence and thus larger placebo effects” (31).

This randomized study aims to fill the gaps in characterizing the efficacy of OLPs in acute pain. We will measure subjective pain ratings and areas of hyperalgesia and allodynia in a well-established experimental pain model (61) and investigate analgesia elicited by OLP injections accompanied by a short evidence-based treatment rationale, as in a previous study (55). To achieve this goal, Part 1 of our study will examine the duration of OLP analgesia and reevaluate the onset and size of the effect. Given the “inert” nature of OLPs, we assume that results of El Brihi et al. (31) concerning OLP dose effects on participant well-being will be generalizable to all outcomes and will, therefore, not evaluate different OLP dosages. Nevertheless and as stated above, multiple doses of placebos and more frequent intake increase effects (59, 60). Furthermore, participants given a choice over their (deceptive) placebo treatment experience enhanced placebo effects (62). For example, participants in control of the time point of administration of their placebo treatment experienced more placebo analgesia than participants without this control [(63); please note: this study investigated deceptive placebos and used conditioning effects]. This leads us to Part 2 of our study in which we will compare outcomes between participants receiving one OLP injection, participants receiving one repetition of the injection at a fixed time point, and participants receiving one repetition of the injection on demand (i.e., the last group will choose if and when they would like to receive the placebo “Booster”). This form of multiple-dose study aims to fill knowledge gaps concerning OLPs.

## 2 Methods and analysis

### 2.1 Study objectives

The primary objective of this study is to further investigate the effect of OLP administration on acute pain in an experimental model of acute pain (simulating wound pain).

#### 2.1.1 Part 1

##### 2.1.1.1 Primary objective

We hypothesize that there will be a difference in the overall subjective pain ratings when comparing subjects receiving a single OLP injection with control subjects receiving no treatment, as suggested by a previous study from our group (55).

##### 2.1.1.2 Further objectives

We hypothesize that there will be a difference in the areas of hyperalgesia and allodynia when comparing subjects receiving a single OLP injection with control subjects receiving no treatment. We hypothesize that the expected difference in subjective pain ratings, hyperalgesia, and allodynia will diminish or even disappear over time as a regular end of dose phenomenon. Insight on the timing of when the effect of the placebo decreases will allow us to determine when to administer a second dose (booster) of OLP (fixed-time booster) in Part 2.

#### 2.1.2 Part 2

##### 2.1.2.1 Primary objective

We hypothesize that there will be a difference in overall subjective pain ratings when comparing subjects receiving a repeated (i.e., two OLP injections) vs. a single OLP injection.

##### 2.1.2.2 Further objectives

In addition, we hypothesize that, in comparison to receiving a repeated OLP injection at a fixed time point, overall subjective pain ratings will be different when subjects receive the second application of OLP on-demand. Moreover, we hypothesize that there will be a difference in areas of hyperalgesia and allodynia when comparing subjects receiving a repeated OLP injection (i.e., two OLP injections) with subjects receiving only a single injection of OLP and when comparing subjects receiving the second application at a fixed time point with subjects receiving the second application on demand.

### 2.2 Study design

#### 2.2.1 Part 1

The first part of this study has been designed as a confirmatory, randomized, two-arm, assessor-blinded cross-over trial in a single center setup at the University Hospital of Basel. We will compare the effects of OLP on acute induced pain intra-individually by delivering the study intervention (OLP) as well as the control intervention (no treatment) to every participant and randomizing only for order of intervention. Thus, the participants will act as their own control.

**FIGURE 1:**
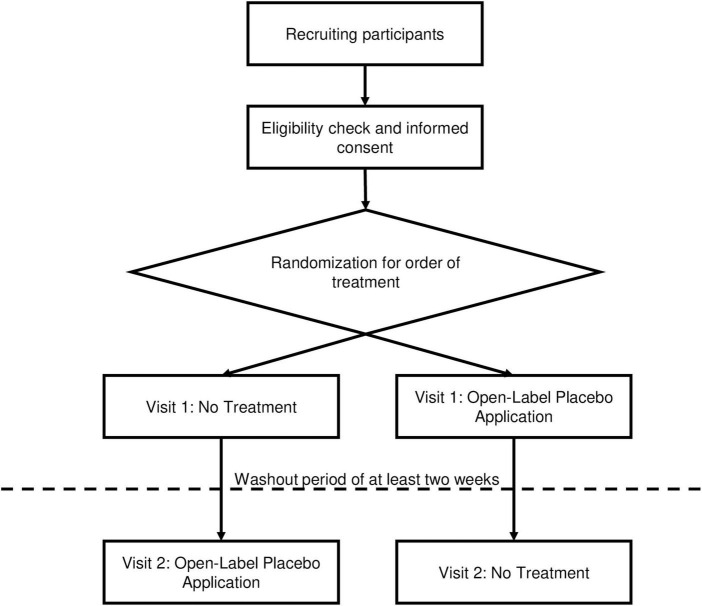
Study design, part 1.

The study design for Part 1 of this study is also shown in Figure 1.

#### 2.2.2 Part 2

The second part of this study has been designed as a proof-of-concept, randomized, controlled, three-arm trial in a single center setup at the University Hospital of Basel. We will compare the effects of an OLP “Booster” inter-individually by analyzing three groups: A fixed-time “Booster” group (who will receive a first OLP at the beginning of the study visit and a second one at a time point derived from Part 1), an on-demand “Booster” group (who will receive a first OLP at the beginning of the study visit and a second one when participants ask for it), and a control group (who will receive only one OLP at the beginning of the study visit). Due to organizational reasons, blinding of the assessor will not be possible in Part 2.

**FIGURE 2:**
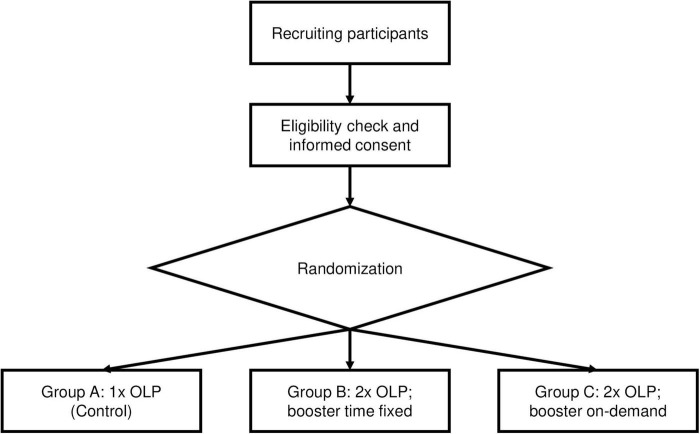
Study design, part 2.

The study design for Part 2 of this study is also shown in Figure 2.

### 2.3 Study population

We will include a total of 36 and 105 healthy adults in Part 1 and Part 2 of our study, respectively. Participants will need to meet all of the following inclusion criteria:

• Healthy volunteers (American Society of Anaesthesiologists Class I or II) aged 18–65 years
• Body Mass Index between 18 and 25 kg/m^2^
• Able to understand the study and the Numeric Rating Scale (NRS)
• Able to give informed consent (IC)

The presence of any of the following exclusion criteria will lead to exclusion of the participant:

• Participation in a previous OLP study; for Part 2, this includes Part 1 of this study
• Regular intake of medications or drugs potentially interfering with pain sensation (analgesics, opioids, antihistamines, calcium and potassium channel blockers, serotonin/noradrenaline reuptake inhibitors, and corticosteroids)
• Neuropathy
• Chronic pain
• Neuromuscular disease
• Dermatological disease (i.e., atopic dermatitis)
• Psychiatric disorder
• Pregnancy/lactation

### 2.4 Study procedures

#### 2.4.1 Recruitment and screening

Participants will be recruited through advertising on the homepage of the University of Basel. For both parts, more participants will be screened to replace possible drop-outs. A balanced gender distribution will be ensured during recruitment.

The volunteers will be contacted by the study team andreceive oral and written information about the study (Supplementary material). Inclusion and exclusion criteria will be assessed and, if eligibility is given, informed consent will be obtained.

In recognition of their efforts, participants will receive standardized financial compensation (i.e., CHF 240.- for the two visits in Part 1 and CHF 120.- for Part 2, respectively) after completion of their study visits.

#### 2.4.2 Study visits

After obtaining informed consent, participants will be given appointments for two study visits and one study visit for Part 1 and Part 2, respectively. The procedures are nearly the same for both study parts and are described below.

##### 2.4.2.1 Briefing

Every participant will be familiarized with the pain scale, the intradermal electrical stimulation model evoking pain, and the hyperalgesia and allodynia examinations. We have chosen not to provide a training session given its invasiveness, including the risk of habituation. Previous studies conducted by our research group using this model have also shown that this is not required (55, 64–67).

##### 2.4.2.2 Experimental setup

First, the study staff will place a venous catheter in the arm (opposite to the one used for electrical stimulation) of each participant.

Second, the intradermal electrical stimulation model to evoke pain will be installed. The model used in this study was first described by Koppert et al. (61) in 2001 and has since been used in numerous studies investigating pain, pain medications, hyperalgesia, and allodynia (55, 61, 64–70). The pain model used in the present study has been shown to provoke stable areas of secondary hyperalgesia to pinprick and touch caused by the activation of mechanoinsensitive C-nociceptors (71), a class of nociceptors shown to be activated electrically, preferentially at high current densities (72, 73).

**FIGURE 3:**
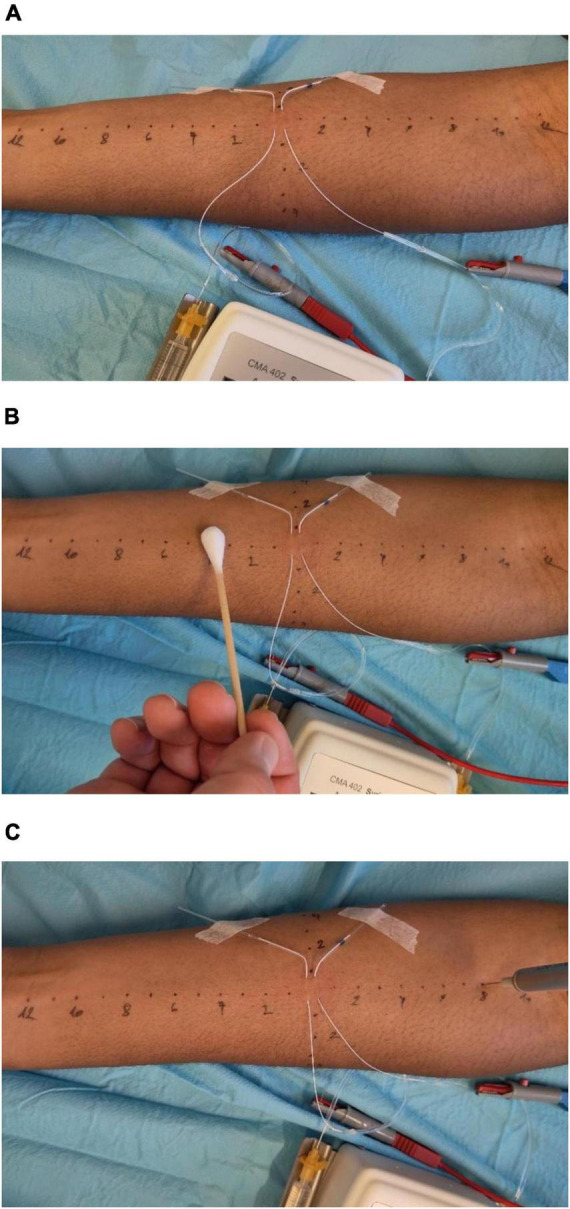
Experimental setup. **(A)** Intradermal electrical stimulation model evoking pain, first described by Koppert et al. (61). **(B)** Assessment of allodynia by using a dry cotton swab. **(C)** Assessment of hyperalgesia using a 256 mN von Frey filament.

In accord with the pain model, two microdialysis catheters with internal stainless-steel wires will be inserted in parallel (5 mm apart) into the intradermal, volar surface of the forearm (not used for venous access) for a length of approximately 10 mm (Figure 3). The catheters will be filled with 0.9% saline set to a continuous flow of 0.4 μl/min, ensured by a syringe pump, to facilitate conduction and to protect the tissue from eventual pH changes by the direct current. To ensure comparability of electrode nociceptor distance regarding subcutaneous tissue thickness after intracutaneous electrode placement only participants with a BMI between 18 and 25 were included. The stainless-steel wires will be attached to a constant current stimulator in combination with a pulse generator. This installation applies monophasic, rectangular electrical pulses 0.5 ms in duration with alternating polarity at a frequency of 2 Hz. The pulse generator pauses the stimulation for 30 s after conducting 500 pulses (approximately 4 min). The current will be increased to target a pain rating of 6 out of 10 on the NRS. Three further adjustments in current will be made every 5 min for the next 15 min to compensate for habituation. The final current will be kept constant until the end of the particular experiment. The total duration of electrical stimulation will be 200 min for Part 1; for Part 2, the duration of stimulation will depend on the results of Part 1. For sure, for ethical, technical and logistic reasons, there will be a limit to the observation time. This time limit will not substantially exceed the duration of stimulation that we know is safe for the skin, i.e., 200 min. At the end of the experiment, the current will be stopped, microdialysis catheters and venous access removed, thereby ending the study visit.

##### 2.4.2.3 Timeline

Tables 1, 2 (for Part 1 and Part 2, respectively) give an overview over the timeline for each study visit.

**TABLE 1:** Schedule for study procedures, part 1.

Possible OLP (intervention only)						x				
Questionnaires	x					x				x
NRS		x	x	x	x	x		x	every 5 min	x
Hyperalgesia		x		x		x		x	every 10 min	x
Allodynia		x		x		x		x	every 10 min	x
Electrical stimulation	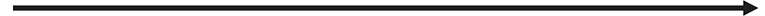									
Time (min)	−5	0	5	10	15	20	25	30	…	200

NRS, numeric rating scale; OLP, open-label placebo.

**TABLE 2:** Schedule for study procedures, part 2.

OLP / OLP Booster (Booster groups only)						x			x*	
Questionnaires	x					x			x*	x
NRS		x	x	x	x	x		x	every 5 min	x
Hyperalgesia		x		x		x		x	every 10 min	x
Allodynia		x		x		x		x	every 10 min	x
Electrical stimulation	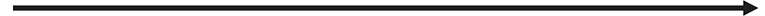									
Time (min)	−5	0	5	10	15	20	25	30	…	**

*either on fixed time point (derived from Part 1; if data is inconclusive, at 100 min) or on-demand.

**measurement time period derived from Part 1. NRS, numeric rating scale; OLP, open-label placebo.

#### 2.4.3 Randomization

As every participant in Part 1 will receive all of the possible treatments, we are only able to randomize the order of the treatments, and thereby control for excitement and habituation effects.

For Part 2, group allocation will be randomized (control group, fixed-time “Booster” group, on-demand “Booster” group).

Randomization will be performed by study staff not involved in study assessments using a random number generator.^1^

#### 2.4.4 Blinding and other methods of minimizing bias

Due to the nature of an OLP study, it is not possible to blind participants. However, in Part 1 of our study, the assessor will be blinded to the intervention participants receive, and the study staff administering the intervention will not be involved in the assessments of the participant under their care.

Blinding of assessors will not be possible in Part 2 due to organizational reasons.

In order to ensure structural equivalence between the study groups and, where possible, blinding of the assessor, all groups will have the same number of visits by study staff giving the intervention. In Part 2, the intervention will be performed on participants by the assessor due to organizational reasons. The assessor will attempt to treat participants from all groups equivalently, although bias cannot be completely ruled out.

### 2.5 Intervention

#### 2.5.1 Experimental intervention

The experimental intervention will consist of two components, similar to our former study (32): a scripted, evidence-based treatment rationale and an OLP injection.

In Part 1, the intervention will be administered 20 min after start of the experiment. In Part 2, there will be two interventions: One 20 min after start of the experiment and one either at a fixed time point (derived from Part 1; or at 100 min if data are inconclusive) or on demand.

##### 2.5.1.1 Treatment rationale

Prior to the administration of the injection, a scripted evidence-based treatment rationale explaining placebo analgesia in pain in general and specifically in OLP will be provided. In the context of OLP treatments, this rationale is believed to be important in order to create a mental state of positive expectation (26), which in combination with the conditioning process elicited by the administration of an inert substance (54) will potentially lead to a placebo effect (i.e., decreased pain perception). A detailed explanation of placebo analgesia and the state of the literature on the effects of OLPs will be given in advance to participants in both groups as part of the written and oral IC procedure (Supplementary material).

The scripted treatment rationale will clearly state the fact that the placebo injections are inactive (inert) and contain only saline (i.e., a saltwater solution). Further, based on previous OLP studies (i.e., 45) it will contain the following discussion points, which have been tailored to refer to the context of the specific placebo analgesia treatment of this study (74):

1. Placebo effects of OLP can be **powerful** in some patients, especially in analgesia.
2. **Treatment expectations** are found to be an important mechanism in placebo analgesia.
3. In response to placebos, the body can **automatically** release **endogenous opioids,** which target the pain that patients experience in association with our experimental pain model.
4. A **positive attitude** is helpful but is not absolutely necessary.

The exact wording of the scripted treatment rationale is available in the Supplementary material.

At the subsequent placebo application in Part 2, the patient will be reminded of the inertness of the injection and that OLPs might help to regulate pain (cf. Supplementary material).

##### 2.5.1.2 OLP injections

Syringes, 5 ml in volume, containing 5 ml of saline 0.9% will be administered intravenously as OLP via the venous access already established at the beginning of the study visit. The study staff administering the saline will ensure that the patient observes the injection.

#### 2.5.2 Control intervention

In Part 1 of the study, no treatment will serve as the control for comparison with the study intervention.

In Part 2, we aim to assess whether a repeated OLP dose is advantageous to a single dose. The control group in this part will receive only a single OLP injection and no repetition. The detailed procedure for the OLP administration is described above (cf. 2.4.1).

As control group participants are likely to experience disappointment (75), they will be reminded of the important role of control group participation after randomization (cf. Supplementary material for the exact wording). Moreover, study staff will strive to treat participants with equal empathy and warmth.

### 2.6 Outcome measures

A timeline of the assessments is shown in Tables 1, 2 for Part 1 and Part 2, respectively.

#### 2.6.1 Part 1

The primary endpoint of Part 1 is the area under the pain curve (AUPC) measured over 3 h according to an 11-point NRS following OLP administration. AUPC will be compared between the interventional visit (after receiving an OLP injection) *vs*. the control (non-interventional) visit.

Secondary endpoints of Part 1 are the differences between subjective pain ratings at each measurement point (NRS score every 5 min after OLP administration), the areas under the curve (AUCs) of area of hyperalgesia and area of allodynia, comparing the interventional *vs*. control (non- interventional) visit.

Hyperalgesia and allodynia will be measured immediately after pain ratings every 10 min as soon as an NRS of 6 is reached for the first time. Pinprick hyperalgesia will be assessed using a 256 mN von Frey filament and allodynia will be determined using a dry cotton swab. Measurements will be conducted from a more distant to a more central site along four orthogonal lines (distal, proximal, lateral, and medial) that will be drawn onto the skin with tick marks indicating each centimeter. Distal and proximal measurements will begin 12 cm from the site of electrical stimulation, and lateral and medial measurements 6 cm from the site. In both cases, the used measuring tool will be moved toward the site of stimulation in 1 cm increments until the subject reports either increased pain sensations from the von Frey filament (hyperalgesia) or an unpleasant “rougher” sensation from the cotton swab (allodynia). To create an area from these linear measurements, we are assuming that the areas are elliptical. The area is calculated using the formula $\frac{1}{4}\,\pi \times D\times d$ (D = proximal/distal diameter; d = medial/lateral diameter). For calculation reasons, we define 1 cm as the smallest value for the diameters.

#### 2.6.2 Part 2

The primary endpoint for Part 2 of our study is the AUPC (after OLP administration, as mentioned above) using the NRS during a time span derived from Part 1. We will compare the AUPCs of participants receiving a single OLP injection, participants additionally administered one repeated OLP injection at the time point derived in Part 1, and participants administered a second OLP injection on-demand.

Secondary endpoints of Part 2 are the same as those for Part 1 of the study.

#### 2.6.3 Other variables of interest

Other variables of interest are:

• Placebo attitudes & beliefs
• Treatment expectations
• Credibility of intervention
• Safety outcomes

##### 2.6.3.1 Placebo attitudes & beliefs

Placebo attitudes and beliefs will be assessed before the setup of the pain model (for Part 1: only during the first visit) using two different scales:

• Placebo attitudes will be assessed using the second item of a questionnaire introduced by Fassler et al. (76), which assesses responders’ attitudes regarding non-specific therapies.
• Participants’ belief in the power of placebos – a measure of the influence of patients’ pre-existing beliefs about placebos related to the effect of OLP – will be assessed using four-item questionnaire developed by Leibowitz et al. (24) and rated using a five-point Likert-scale. In line with the procedure proposed by Beaton et al. (77), we have translated the scale from English to German using back translation for verification.

##### 2.6.3.2 Treatment expectations

Treatment expectations will be assessed prior to the intervention (OLP alone and OLP & “Booster” for Parts 1 and 2, respectively) by the study staff administering the intervention. The assessment will be done using two items (“Expected Benefits,” “Desired Benefits”) of the Treatment Expectation Questionnaire developed by Alberts et al. (78) and translated into German (cf. Supplementary material).

##### 2.6.3.3 Credibility of intervention

If receiving an OLP injection, participants will be asked about the credibility of the OLP intervention at the end of the study visit (for details cf. Supplementary material).

#### 2.6.4 Safety outcomes

Due to the “inert” nature of placebos, no specific adverse events are expected. However, safety endpoints including the patient’s well-being, pain, skin irritation/infection, and allergic reactions/hypersensitivity will be noted. Thereby, we will assess adverse events of the pain model and/or the infusion of fluids through the venous access.

### 2.7 Data collection, management and storage

For data collection and management of participants’ responses at study visits, the secure web application REDCap (79) will be used as an electronic case report form. The system is hosted by the Center for Scientific Computing of the University of Basel (sciCore) and is password protected. Only authorized personal will be able to enter the system to view and/or edit data. Double data entry is performed in REDCap to digitalize all source documents, and all data entries are de-identified. Regular back-ups of study data take place, and back-ups are stored on secure webservers of the University Hospital Basel.

### 2.8 Sample size

For Part 1, we estimated how many participants are needed to achieve an 80% power to detect a difference of 45 in the AUPC between the interventional visit and the control visit. Based on data from our previous study (55), we assumed a standard deviation of 67 for the differences in the AUPC. The assumed difference and standard deviation of the difference correspond to a Cohen’s d of 0.7. Calculations were done assuming a two-sided alpha of 5% and using a two-sample *t*-test for comparison. The number of participants needed for Part 1 is 36.

The sample size for Part 2 was determined with respect to the comparison of the combined “Booster” (i.e., fixed-time and on-demand) groups *vs*. the control group. Including 105 participants allows to estimate a difference in the AUPC of 45 between the “Booster” groups and the control group and a difference of the AUPC of 51 between the fixed-time and on demand group with a power of 80%.We assume a standard deviation of 76 for the AUPC in all groups, which corresponds to a Cohen’s d of 0.6. Calculations were done assuming a two-sided alpha of 5% and using a two-sample *t*-test for comparison.

### 2.9 Statistical analysis

#### 2.9.1 Primary analyses

In Part 1, the effect of the OLP on the AUPC will be tested using a two-sample *t*-test. In order to account for additive period effects, the estimator of the treatment effect will be built as follows: The mean of the difference in the AUPC between the first and the second visit will be calculated for all patients who receive the placebo in the second visit. Then, the mean of the difference in the AUPC between the first and the second visit will be calculated for all patients who receive the placebo in the first visit. Then, the difference of the first and the second mean will be calculated and divided by two.

In Part 2, the AUPCs of the three groups will be compared as follows: First, the mean of the AUPC in the “Booster” groups (fixed-time and on-demand) will be compared to the mean of the AUPC in the control group using a two-sample *t*-test. If the test yields a significant difference, the mean of the AUPC in the fixed-time “Booster” group will be compared to the mean of the AUPC in the on-demand “Booster” group using a two-sample *t*-test.

#### 2.9.2 Secondary analyses

In order to establish a suitable time point for administering the “Booster” of the placebo, the time course of the NRS pain score will be investigated. For each participant, the difference in the NRS pain score between the interventional visit and control visit will be calculated. We will then fit a broken stick mixed-effect model (80) assuming normal distribution of the error, including the type of visit as a fixed factor, and an identifier of the participant as a random factor. The model will account for random intercepts only. The optimal breakpoint will be selected based on the residual standard error. We will also model the time course of NRS pain with Gaussian process regression (81).

Furthermore, the AUC of areas of hyperalgesia and allodynia will be compared between the two visits in Part 1 and between the three arms in Part 2, applying the same analysis as described for the primary endpoint. The development of NRS pain score, hyperalgesia, and allodynia over time will be presented graphically.

The influence of the variables listed in “other variables of interest” (c.f. 2.6.3.) on the outcome will be analyzed by means of linear regression models with the difference between the intervention and the control visit in the area under the curve of pain, allodynia and hyperalgesia as the outcome and the arm and the score of the respective question as predictors.

#### 2.9.3 Sensitivity analyses

In order to further examine the effect of OLP application on subjective pain ratings, the following sensitivity analyses will be conducted:

1. In order to be able to directly compare the results with previous studies (55), we will repeat the primary analysis for a restricted AUPC using only the first 90 min of measurement.
2. We will explore the relationship between the outcome and the time between the two visits by fitting a linear regression model with the difference in the AUPC as the outcome and the arm, the time between the visits and the interaction term arm × time between visits as predictors.
3. We will examine the relationship between the difference in the AUPC and several demographic covariates namely (i) age (ii) sex, and (iii) level of education. To this end, we will fit linear regression models with the difference in the AUPC as the outcome and the arm, the covariate and the interaction term arm × covariate as predictors.

These sensitivity analyses are exploratory and not confirmatory, hence no hypotheses are tested and *p*-values will be interpreted as continuous measures that inform the generation of new hypotheses worthy of further investigation.

#### 2.9.4 Interim analyses

No interim analysis is planned.

### 2.10 Monitoring

The study will be monitored for quality and regulatory adherence by an independent monitor of the University Hospital of Basel. The monitor will verify the qualifications of the investigators and study team members and control for sound and appropriate documentation. In addition, monitoring visits will serve to ensure that:

• The study is being conducted according to the study protocol and within the specified time frame.
• Data are collected accurately and are completely documented in REDCap, and the source documents.
• The interventional medication (placebo injections) are being correctly prepared, dispensed and accounted for.
• Side effects are being correctly defined, assessed, and documented.

## 3 Discussion

Placebo effects have already been observed for a long time. Since Beecher’s often-quoted 1955 study (82), several publications have reported on placebos and placebo effects. Hrobjartsson and Gotzsche (13) provide a nice overview. Significant effects on various conditions have been found, especially patient-reported outcomes, such as pain (13, 16). However, ethical concerns about the deceptive use of placebos have grown (18–20). Therefore, OLPs have been proposed (21) as a means of using placebos in an ethical manner. There is a rising body of knowledge concerning OLPs, and they have been proven to be effective in various clinical conditions including chronic pain. Charlesworth et al. (56) and von Wernsdorff et al. (57) provide an overview of these effects.

Although a well-known challenge, pain management remains difficult in many situations. Therapy options are limited, and positive effects are often diminished by contraindications, side effects, and adverse events (e.g., 2–8). This illustrates the need for new broadly applicable treatments, preferably with few or no side effects, which could augment existing multimodal analgesic concepts (7). OLPs potentially fit this description given the near absence of contraindications and few if any side effects, which are likely avoidable and usually present as nocebo effects (11, 17). Already, a small amount of evidence supports the use of OLPs in acute pain (25–29, 47, 49, 52, 53). However, for clinical implementation, the efficacy of these interventions need to be characterized to a greater extent. This study contributes to this by measuring the duration of OLP analgesia in acute pain. To our knowledge, this is the first study investigating whether repeated OLP doses are more beneficial than a single-dose and when repeated dosing should occur.

A strength of this study is the cross-over design of Part 1, in which participants serve as their own control, thereby enabling comparison between the interventional and control group. Part 2 does not have a cross-over design. Rather, participants are randomized into three different groups, establishing the classic form of randomized controlled trial. In addition, all procedures will be highly standardized, as the same investigators and respective study staff will be interacting with all participants included in each study part. Further strengths include the use of a well-established pain model (61) and the long 200-min measurement period. Finally, in this experimental setting, the assessor will perform measurements every 5 min, which should result in no missing data, contrary to what would be expected for experiments utilizing self-reported questionnaires.

This study also has a few limitations. As this is an experimental study with highly standardized procedures, results will have to be transferred to clinical application with caution. The placebo effect could be altered in clinical populations with possible interfering variables such as comorbidities [particularly if the cognition is impaired (83)], concomitant medication, (negative) emotions (84) or deviant procedures [e.g., no sufficient OLP rationale (26)]. In summary, with this study, we strive to find hypotheses concerning OLPs, which can later be translated to and tested in clinical populations (32).

Three further factors compromise generalizability. First, the population will be recruited through an advertisement on the homepage of the local university, which may lower the diversity of the study population. Second, some bias cannot be ruled out. Due to the nature of OLPs, blinding of participants is not possible. In addition, due to organizational reasons, even the assessor will not be blinded to the intervention in Part 2. Furthermore, it could also be argued, that not providing an interventional rationale to the control group in Part 1 could result in a difference in patient-provider relationship between interventional and control group. Third, for Part 1, we assume that there are no carry-over effects (i.e., we assume that the treatment sequence will not modify the treatment effect). Specifically, while we account for period effects (e.g., in the form of learning to better deal with pain from the first to the second treatment period), we assume that this will not depend on whether the participant received the placebo or the control intervention during the first visit.

In addition, the measurement period could also be seen as a limitation: Although with 200 min it is quite long, and – to our knowledge – longer than the observation time of previous comparable studies, it could still be insufficiently long to fully observe OLP analgesia for acute pain.

In conclusion, although the study faces a few limitations, we are convinced that it has been designed in a way that will extend the knowledge about the use of OLPs in the treatment of acute pain. In particular, the duration of OLP analgesia and possible “Booster” effects will need to be evaluated before implementation to clinical practice is possible. Therefore, this study will add another step towards clinical implementation of a new, broadly applicable treatment for acute pain with few to no contraindications or side effects.

## 4 Ethics and dissemination

This study is conducted in compliance with the protocol, the current version of the Declaration of Helsinki (85), the ICH-GCP (86), the HRA (87) as well as other locally relevant legal and regulatory requirements.

### 4.1 Confidentiality

Data will be handled confidentially, be protected and encoded. Participants’ confidentiality will be maintained at all times. Direct access to source documents will be permitted for purposes of monitoring, audits, and inspections. However, medical secrecy will be respected, and the identity of the participants will not be divulged at any time. Investigators, study team members, and statisticians will have access to the protocol, datasets, statistical codes, and other relevant data during and after study period.

### 4.2 Dissemination policy

The results of the planned analyses will be published in a peer-reviewed journal. Talks at conferences and other occasions (e.g., teaching) are also planned.

## Ethics statement

The protocol of this study was approved by “Ethikkomission Nordwest- und Zentralschweiz” (BASEC 2023-00296) on March 15, 2023. Trial and protocol design were developed according to the Standard Protocol Items: Recommendations for Interventional Trials (SPIRIT) guidelines (74). The studies were conducted in accordance with the local legislation and institutional requirements. The participants provided their written informed consent to participate in this study.

## Author contributions

MdL, ML, JG, WR, and TS contributed to the conception and design of the study. MdL, ML, and TS drafting of the manuscript. All authors contributed to the critical revision, proofreading, and approving of the submitted version.

## Footnotes

1
www.randomizer.org
