# Supplementary material for: Boosting open-label placebo effects in acute induced pain in healthy adults (BOLPAP-study): study protocol of a randomized controlled trial
Source: Front Med (Lausanne). 2024 Feb 14;11:1238878. doi: 10.3389/fmed.2024.1238878 (PMC10900763; doi:10.3389/fmed.2024.1238878)
Supplement: Supplementary file 2 [file Data_Sheet_2.PDF]

Universitätsspital Basel

Universität Basel
Fakultät für Psychologie

Anfrage zur Teilnahme an medizinischer Forschung

Studientitel: Boosting Open-Label Placebo Effects in Acute Induced Pain in Healthy Adults

Laienverständlicher Titel: Wirkdauer und Boost-Effekte von offen verabreichten Placebos bei akuten induzierten Schmerzen

Sehr geehrte Dame, sehr geehrter Herr

Hiermit möchten wir Sie über unsere Studie informieren und Sie anfragen, ob Sie daran teilnehmen möchten. Denn bevor eine neue Interventionsmethode von Ärztinnen und Ärzten angewendet werden darf, muss erforscht werden, wie diese Interventionsmethode wirkt.

Eine solche Forschung nennen wir eine **klinische Studie**. In dieser Studie wollen wir herausfinden, welchen Effekt offen verabreichte Placebos bei gesunden Versuchspersonen auf akute Schmerzen haben. Sie sind gesund. Deshalb fragen wir Sie an, ob Sie an dieser Studie teilnehmen möchten.

Ihre Teilnahme ist freiwillig. Die folgende **Studieninformation** soll Ihnen bei der Entscheidung helfen. Alle Fragen zur Studienteilnahme können Sie im **Gespräch mit der Prüfärztin / dem Prüfarzt** stellen. So nennen wir die Ärztinnen und Ärzte, die für eine Studie verantwortlich sind und die Sie im Rahmen dieser Studie betreuen. Wenn Sie teilnehmen wollen, unterzeichnen Sie bitte am Ende die **Einwilligungserklärung**. Mit Ihrer Unterschrift bestätigen Sie, dass Sie die Studieninformation gelesen und verstanden haben. Wenn Sie etwas nicht verstehen, fragen Sie bitte die Prüfärztin / den Prüfarzt.

Die Studieninformation und Einwilligungserklärung bestehen aus vier Teilen:

Teil 1 **Das Wichtigste in Kürze**
Teil 2 **Darum geht es im Detail: Informationen zur Studie**
Teil 3 **Datenschutz und Versicherungsschutz**
Teil 4 **Einwilligungserklärung**

Wenn Sie **Teil 1** lesen, dann erhalten Sie einen Überblick über die Studie. In **Teil 2** erklären wir Ihnen den ganzen Ablauf und Hintergrund der Studie im Detail. **Teil 3** enthält die Informationen zum Daten- und Versicherungsschutz. Mit Ihrer Unterschrift am Ende des Dokuments, **Teil 4**, bestätigen Sie, dass Sie alles verstanden haben und mit der Teilnahme einverstanden sind.

Diese Studie wird von der Klinik Anästhesiologie des Universitätsspitals Basel in Kooperation mit der Fakultät für Psychologie der Universität Basel veranlasst. Die Klinik Anästhesie des Universitätsspitals Basel ist dabei der Sponsor. Der Sponsor verantwortet, leitet und finanziert eine Studie.

Im Rahmen dieser Studie ist für Sie zuständig:

Name	Dr. med. Tobias Schneider
Adresse	Universitätsspital Basel<br>Abteilung Schmerztherapie, Klinik Anästhesiologie<br>Spitalstrasse 21, 4031 Basel
Telefon	+41 61 328 65 43 / +41 61 265 25 25 (24h Notfallkontakt über DA Anästhesie)
E-Mail	tobias.schneider@usb.ch

Teil 1:
Das Wichtigste in Kürze

1. Warum führen wir diese Studie durch?

In dieser Studie untersuchen wir, wie sich die offene (für den Teilnehmer ersichtliche/ offen kommunizierte) Applikation eines Placebos (medizinisch inaktive Substanz) auf einen im Modellversuch erzeugten Schmerzreiz auswirkt.

Wir erhoffen uns von dieser Studie nützliche Informationen über ein mögliches Potential der offenen Placebo Applikation bei akuten Schmerzen in der klinischen Praxis.

In **Kapitel 4** erfahren Sie mehr zum wissenschaftlichen Hintergrund der Studie.

2. Was müssen Sie tun, wenn Sie teilnehmen?

Die Teilnahme an dieser Studie dauert für Sie etwa drei Tage. Wir werden Sie für eine Studienvisite einladen, welche etwa viereinhalb Stunden dauert. Vorgängig werden Sie ein Gespräch mit der Prüfärztin bzw. dem Prüfarzt haben.

Der Hauptteil der Studienvisite besteht darin, dass dünne Drähte durch die Haut am Unterarm gelegt werden und ein Strom über diese Drähte kontinuierlich über ca. 200 Minuten appliziert wird. Während dieser Zeit werden verschiedene Schmerztests durchgeführt. Wir werden zudem eine offene Placebo (medizinisch inaktive Substanz) Gabe durchführen. Je nach Gruppe, zu der Sie zugeteilt werden, erhalten Sie eine oder zwei offene Placebo-Gaben.

Diese Studie ist eine randomisierte, kontrollierte Studie. Dies bedeutet, dass mehrere verschiedene Interventionen getestet werden. Wenn Sie sich entscheiden, teilzunehmen, werden Sie zufällig einer Studiengruppe zugeteilt. Dies bedeutet, dass Sie entweder eine (Kontrolle) oder zwei (Intervention) offene Placebo-Gaben erhalten werden.

In **Kapitel 5** erfahren Sie mehr zum Ablauf und Vorgehen der Studie.

3. Welcher Nutzen und welches Risiko sind mit der Teilnahme verbunden?

Nutzen

Sie haben keinen direkten Nutzen durch die Teilnahme an der Studie. Es ist aber möglich, dass Sie mit Ihrer Teilnahme künftigen Patientinnen und Patienten helfen. Der Nutzen für die Allgemeinheit liegt im zusätzlichen Erkenntnisgewinn über die Wirkung von offen verabreichten Placebos auf akute Schmerzen. Die gewonnenen Informationen können für den möglichen klinischen Einsatz wertvoll sein.

Für Ihren Aufwand bezüglich der Studienteilnahme erhalten Sie eine finanzielle Entschädigung (vgl. **Kapitel 7**).

Risiko

Offen verabreichte Placebos sind in der Schweiz noch nicht für die Behandlung akuter Schmerzen zugelassen. Die als Placebo verwendete Substanz (Kochsalzlösung / NaCl 0.9%) ist in der Schweiz jedoch zugelassen. Wird die Substanz in so kleinen Mengen wie in dieser Studie verwendet und intravenös verabreicht, sind keine Nebenwirkungen zu erwarten.

Bzgl. des eingelegten Venenverweilkatheters (zur intravenösen Verabreichung des Placebos) besteht das Risiko von Blutergüssen, Blutungen oder Schwellungen an der Einstichstelle. Selten kann es zu einer Infektion an der Einstichstelle kommen.

Bzgl. der eingelegten dünnen Drähte bestehen folgende Risiken:

- Lokale Hautirritation
- Milde Infektion

Dies wurde jedoch sehr selten beobachtet und heilt in der Regel nach wenigen Tagen ab.

Die verwendeten Stromstärken (0.1 – ca. max. 30 mA) zeigen keine den ganzen Körper betreffende Wirkung und sind für die Nervenfasern und die Haut nicht schädlich.

In **Kapitel 6** finden Sie weitere Informationen zu Risiken und Belastungen.

Teil 2:
Darum geht es im Detail: Informationen zur Studie

4. Der wissenschaftliche Hintergrund der Studie

4.1 Hintergrund: Warum führen wir diese Studie durch?

Schmerzen sind ein häufiges Symptom bei Patientinnen und Patienten, insbesondere im Spital. Typischerweise werden Schmerzen mit Basisschmerzmitteln wie Paracetamol, nicht-steroidalen Antirheumatika und Metamizol sowie stärkeren Schmerzmitteln (Opiaten) behandelt. Deren Einsatz wird aber durch Anwendungseinschränkungen und Nebenwirkungen begrenzt. Deshalb wären zusätzliche, breit einsetzbare und nebenwirkungsarme Massnahmen zur Behandlung von Schmerzen sehr wertvoll.

Wir untersuchen daher in dieser Studie, ob offen verabreichte Placebos (medizinisch inaktive Substanzen) eine wirksame Schmerzlinderung auslösen können.

Es gibt schon Forschung am Menschen zur Wirkung von Placebos. Bisherige Studien am Menschen haben gezeigt, dass diese eine nachweisbare und klinisch bedeutsame Wirkung bei chronischen und akuten Schmerzzuständen haben. Wir wissen auch, dass Placebos auch wirken können, wenn sie offen verabreicht werden; das heisst, die Patientinnen und Patienten wissen, dass sie eine medizinisch inaktive Substanz erhalten. Diese offen verabreichten Placebos zeigten z.B. bei chronischen Schmerzzuständen, wie beim Reizdarmsyndrom oder chronischen Rückenschmerzen, eine Effektivität in der Schmerztherapie. Es wird dabei einerseits davon ausgegangen, dass der Körper automatisch auf die Einnahme von Medikamenten reagiert und die dabei ausgelöste Schmerzlinderung durch die Freisetzung körpereigener Opioide vermittelt wird. Das heisst, der Körper produziert selber Stoffe, sogenannte Endorphine, die auf dieselbe Art und Weise wie sehr starke Schmerzmittel im Körper wirken. Andererseits wird davon ausgegangen, dass auch schon alleine durch die Zuwendung und Fürsorge einer Ärztin bzw. eines Arztes eine Besserung eintreten kann.

Die Wirksamkeit von offen verabreichten Placebos bei akuten Schmerzen ist bisher nur wenig untersucht. Insbesondere haben wir zu wenige Daten bzgl. wiederholter Anwendung (Boost-Effekte) dieser offen verabreichten Placebos. Diesen Aspekt möchten wir nun mit dieser Studie untersuchen, und damit einen weiteren Schritt in Richtung der Zulassung von offen verabreichten Placebos für akute Schmerzen machen. Denn erst wenn die Wirksamkeit einer Interventionsmethode genügend wissenschaftlich untersucht und erwiesen ist, kann sie in der Schweiz zugelassen und eingesetzt werden.

4.2 Aufbau der Studie: Wie gehen wir vor?

Patientinnen und Patienten, die innerhalb der Einschlusskriterien liegen und keines der Ausschlusskriterien erfüllen, können an der Studie teilnehmen. Die Kriterien sind wie folgt definiert:

Einschlusskriterien:

- Gesunde (American Society of Anaesthesiologist’s Kategorie I oder II) 18 – 65-jährige Menschen
- BMI zwischen 18 und 25kg/m^2
- Verstehen der Grundzüge der Studie sowie der Anwendung der Numerischen Schmerzrating-Skala (NRS)
- Fähig, das schriftliche Einverständnis zu geben

Ausschlusskriterien:

- Teilnahme an einer früheren Studie zur Erforschung von offen verabreichten Placebos
- Regelmässige Einnahme von Medikamenten oder Drogen, die potentiell einen Einfluss auf Schmerzempfindung und/oder –wahrnehmung haben wieSchmerzmittel, Antihistaminika («Antiallergika»), Calcium- und/oder Kaliumkanalblocker (Einsatz u.a. gegen Bluthochdruck, Herzrhythmusstörungen, Diabetes mellitus, Multiple Sklerose), Antidepressiva (SNRI, SSRI), Cortison-haltige Medikamente
- Neuropathie
- Chronische Schmerzen
- Neuromuskuläre Erkrankungen
- Dermatologische Erkrankungen (z.B. atopische Dermatitis)
- Psychiatrische Erkrankungen
- Schwangerschaft / Stillzeit

In unserer Studie werden die Teilnehmenden zufällig einer Intervention zugeteilt. Dies ist wichtig, um verlässliche Ergebnisse der Studie zu erhalten. Man nennt dies Randomisierung. Die Interventionen sind:

- **Booster-Intervention:** An diesem Termin erhalten die Teilnehmenden innerhalb des Messzeitraums zwei intravenöse Injektionen mit einem Placebo (Kochsalzlösung, NaCl 0.9%) und eine dazugehörige Erklärung.
- **Kontroll-Intervention:** An diesem Termin erhalten die Teilnehmenden eine intravenöse Injektion mit einem Placebo (Kochsalzlösung, NaCl 0.9%) und eine dazugehörige Erklärung, aber keine Wiederholung der Placebo-Gabe. Dieser Termin ist wichtig, um die Wirkung der Booster-Intervention mit dem Verlauf des Schmerzempfindens bei einmaliger Placebo-Gabe vergleichen zu können.

Durch die Randomisierung können wir objektiv beurteilen, wie gut offen verabreichte Placebos wirklich wirken.

4.3 Regelungen zur wissenschaftlichen Forschung mit Menschen

Wir machen diese Studie so, wie es die Gesetze in der Schweiz vorschreiben (Humanforschungsgesetz, Datenschutzgesetze). Ausserdem beachten wir alle international anerkannten Richtlinien. Die zuständige Ethikkommission hat die Studie geprüft und bewilligt.

Eine Beschreibung dieser Studie finden Sie auch auf der Internetseite des Bundesamtes für Gesundheit unter www.kofam.ch unter der SNCTP-Registriernummer SNCTP000005470 oder der BASEC-Nummer 2023-00296.

5. Ablauf der Studie

5.1 Was müssen Sie tun, wenn Sie an der Studie teilnehmen?

Die Teilnahme an der Studie ist freiwillig und dauert ca. drei Tage. Sie müssen sich an den Ablaufplan halten (→ **Kapitel 5.2**) und auch an alle Vorgaben, die Ihre Prüfärztin / Ihr Prüfarzt macht.

Sie müssen Ihre Prüfärztin / Ihren Prüfarzt informieren,

- wenn sich Ihr Gesundheitszustand ändert, z. B. wenn es Ihnen schlechter geht oder wenn Sie neue Beschwerden haben; dies gilt auch, wenn Sie die Studie vorzeitig abbrechen (→ Kapitel 5.3 und 5.4);
- wenn Sie gleichzeitig bei anderen Ärztinnen oder Ärzten behandelt werden und/oder Medikamente (auch Medikamente der Komplementär- und Alternativmedizin) einnehmen.

Sie müssen ausserdem Folgendes beachten:

- Sie müssen während der Teilnahme das Eintreten einer Schwangerschaft wirksam verhüten (→ **Kapitel 5.5**).
- Sie dürfen während der Teilnahme keine Drogen und/oder die Schmerzwahrnehmung verändernde Substanzen einnehmen. Bei Unsicherheit diesbezüglich wenden Sie sich bitte an die Prüfärztin bzw. den Prüfarzt.

5.2 Was passiert bei den Terminen?

Im Verlauf Ihrer Teilnahme kommen Sie einmal zu einem Studienbesuch zu uns, der ungefähr viereinhalb Stunden dauert. Vor diesem Termin werden in einem Gespräch nach einer ausführlichen Erklärung durch die Prüfärztin bzw. den Prüfarzt allfällige Fragen beantwortet, die Ein- und Ausschlusskriterien überprüft und anschliessend die Einverständniserklärung eingeholt.

Beim Studientermin machen wir Folgendes:

- Wir beantworten Ihre Fragen.
- Wir überprüfen die Ein- und Ausschlusskriterien; insbesondere wird, um eine Schwangerschaft bei Frauen im gebärfähigen Alter auszuschliessen, vor Studiendurchführung ein Schwangerschaftstest (mit Urinprobe) durchgeführt.
- Wir legen an einem Arm einen Venenverweilkatheter und schliessen eine Infusion (mit Kochsalzlösung, NaCl 0.9%) an.
- Wir legen unter Einhaltung der höchsten Hygiene- und Sicherheitsstandards zwei dünne Drähte durch die oberflächliche Haut an der Innenseite des nicht für die Venenverweilkanüle verwendeten Unterarms. Über die Drähte werden Stromreize appliziert, bis ein mittlerer Schmerz entsteht.
- In regelmässige Abständen werden nun über 200 Minuten Fragen bzgl. der Schmerzintensität gestellt und zwei weitere Untersuchungen zur Beurteilung der Schmerzqualität durchgeführt.
- Es wird zudem eine der zwei Interventionen (vgl. **Kapitel 4.2**) durchgeführt.

Wir vereinbaren den Termin gemeinsam mit Ihnen. Der Termin kann nicht einfach verschoben werden. Wir bitten Sie, uns schnell zu informieren, wenn Sie den Termin trotzdem aus wichtigen Gründen verschieben müssen.

5.3 Wann endet die Teilnahme an der Studie?

Für Sie dauert die Teilnahme etwa drei Tage und endet nach der Studienvisite. Sie können Ihre Teilnahme jederzeit auch früher abbrechen (→ **Kapitel 5.4**). Sie müssen nicht erklären, warum Sie nicht mehr teilnehmen möchten. Wenn Sie selbst Ihre Teilnahme früher beenden möchten, sprechen Sie bitte mit Ihrer Prüfärztin bzw. Ihrem Prüfarzt. Dies hat für Sie keinerlei negative Konsequenzen.

Wenn Sie die Studie früher abbrechen, bitten wir Sie, Ihren Prüfarzt bzw. Ihre Prüfärztin weiterhin zu informieren, wenn sich Ihr Gesundheitszustand ändert, z.B. wenn es Ihnen schlechter geht oder wenn Sie neue Beschwerden haben. Wenn Ihre Teilnahme vorzeitig endet, werden wir die bis dahin erhobenen Daten noch für die Studie auswerten.

Es kann auch sein, dass wir Sie bitten müssen, die Studie frühzeitig zu beenden. Das ist zum Beispiel der Fall, wenn sich bei Ihnen zwischen dem Vorgespräch und dem Studientermin neu ein Ausschlusskriterium ergibt.

5.4 Was passiert, wenn Sie nicht teilnehmen möchten?

Wenn Sie nicht interessiert sind und/oder mit den Risiken nicht einverstanden sind, werden Sie nicht in die Studie eingeschlossen. Dies hat für Sie keinerlei negative Konsequenzen.

5.5 Schwangerschaft

Es gibt noch keine Daten über die Wirkung von oberflächlich applizierten Stromreizen auf das ungeborene Kind.

Für Frauen, die schwanger werden können

Sie dürfen während Ihrer Teilnahme an der Studie nicht schwanger werden. Folglich wird bei Frauen im gebärfähigen Alter zu Beginn jeder Studienvisite ein Schwangerschaftstest (im Urin) durchgeführt. Zudem müssen Studienteilnehmerinnen während der Studie eine wirksame Verhütungsmethode anwenden. Sollten Sie bereits eine hormonelle Verhütungsmethode (z.B. die «Pille» oder eine Hormonspirale) verwenden, empfehlen wir Ihnen, diese auch während der Studie zu nutzen und zusätzlich Kondome anzuwenden. Sollte dies nicht der Fall sein, empfehlen wir Ihnen eine der folgenden Möglichkeiten zur Verhütung: Kondom, Pessar, Verhütungskappe, Diaphragma oder Schwamm, jeweils kombiniert mit Spermizidgel.

Wenn Sie im Verlauf der Studie trotzdem schwanger werden, müssen Sie das sofort Ihrer Prüfärztin / Ihrem Prüfarzt sagen und dürfen nicht weiter an der Studie teilnehmen. Da wir nicht von einem Einfluss von applizierten Stromreizen auf eine Schwangerschaft ausgehen, werden keine Verlaufskontrollen geplant.

Wenn Sie stillen, dürfen Sie ebenfalls nicht an der Studie teilnehmen.

Für Männer

Es ist bislang nicht ausreichend bekannt, ob das in dieser Studie verwendete Schmerzmodell möglicherweise Ihre Spermien schädigen kann. Sie müssen daher Ihre Partnerin(nen) darüber informieren, dass Sie an dieser Studie teilnehmen.

Während der Teilnahme an der Studie dürfen Sie nur mit Kondomen Geschlechtsverkehr haben. Weil das Kondom als alleinige Verhütungsmethode aber nicht sicher genug ist, muss Ihre Partnerin (bzw. müssen Ihre Partnerinnen) zusätzlich eine wirksame Verhütungsmethode anwenden (siehe oben).

Wenn Ihre Partnerin im Verlauf der Studie trotzdem schwanger wird, müssen Sie das sofort Ihrem Prüfarzt / Ihrer Prüfärztin sagen und dürfen nicht weiter an der Studie teilnehmen. Da wir nicht von einem Einfluss von applizierten Stromreizen auf eine Schwangerschaft ausgehen, werden keine Verlaufskontrollen geplant.

6. Risiken, Belastungen und Nebenwirkungen

6.1 Welche Risiken und Belastungen können auftreten?

Es gibt Risiken und Belastungen bei der Teilnahme an dieser Studie, wie bei jeder medizinischen Intervention. Sie finden nachfolgend eine Liste der häufigsten und schwersten Risiken. Viele Nebenwirkungen sind medizinisch behandelbar.

Für die Einlage der Drähte in die oberste Hautschicht wird es einen Stich geben, der etwas schmerzhaft sein kann. Um dies zu lindern, wir die Hautoberfläche vorgängig mit Eis gekühlt. Durch das Legen der Drähte kann es dort zu lokalen Hautirritationen und/oder Infektionen kommen. Dies wurde jedoch sehr selten beobachtet und heilt in der Regel nach wenigen Tagen ab, sodass keine Rückstände ersichtlich oder spürbar sind.

Die verwendeten Stromstärken von ca. 0.1 – maximal 30 mA zeigen keine systemische Wirkung («Ganzkörperwirkung») und sind für die Nervenfasern und die Haut nicht schädlich.

Bzgl. des eingelegten Venenverweilkatheters (zur intravenösen Verabreichung des Placebos) besteht das Risiko von Blutergüssen, Blutungen oder Schwellungen an der Einstichstelle. Selten kann es zu einer Infektion an der Einstichstelle kommen.

Offen verabreichte Placebos sind in der Schweiz noch nicht für die Behandlung akuter Schmerzen zugelassen. Die als Placebo verwendete Substanz (Kochsalzlösung / NaCl 0.9%) ist in der Schweiz jedoch zugelassen. Wird die Substanz in so kleinen Mengen wie in dieser Studie verwendet und intravenös verabreicht, sind keine Nebenwirkungen zu erwarten.

7. Finanzierung und Entschädigung

Diese Studie wird vom Sponsor veranlasst und bezahlt. Die verwendeten Materialien und Geräte werden kostenlos vom Universitätsspital Basel zur Verfügung gestellt.

Die beteiligten Forschenden haben keinen unmittelbaren finanziellen Vorteil an der Durchführung dieser Studie.

Wenn Sie bei dieser Studie mitmachen, bekommen Sie folgende Entschädigung:

- Versuchstag 1, ca. viereinhalb Stunden: CHF 120.-

Im Falle eines unerwarteten, vorzeitigen Abbruchs der Studie während eines Versuchs erhalten Sie trotzdem eine Entschädigung. Diese ist an den Aufwand angepasst und wird mit CHF 30.- pro Stunde berechnet.

Durch die Teilnahme an der Studie entstehen keine zusätzlichen Kosten für Sie oder für Ihre Krankenkasse.

8. Ergebnisse aus der Studie

Es gibt Ergebnisse, die Sie selbst betreffen. Diese Ergebnisse teilt Ihnen Ihre Prüfärztin / Ihr Prüfarzt mit. Es gibt auch Zufallsbefunde. Zufallsbefunde sind „Begleit-Ergebnisse“, die nicht beabsichtigt sind. Wir informieren Sie, wenn diese Zufallsergebnisse relevant sind für Ihre Gesundheit.

Wir informieren Sie zum Beispiel, wenn wir zufällig eine Erkrankung feststellen, von der Sie noch nichts wissen und die wir behandeln können. Wir informieren Sie auch, wenn wir ein Risiko für eine Erkrankung finden, die man durch vorbeugende Massnahmen verhindern kann. Wenn Sie *nicht* informiert werden wollen, besprechen Sie das bitte mit Ihrer Prüfärztin / Ihrem Prüfarzt.

Es gibt auch die Gesamtergebnisse der Studie, die aus den Daten von allen Teilnehmenden kommen. Dazu gehört zum Beispiel, dass wir mehr wissen über offen verabreichte Placebos in der Akutschmerztherapie (→ **Kapitel 4.1**). Diese Ergebnisse betreffen Sie und Ihre Gesundheit nicht direkt. Ihre Prüfärztin / Ihr Prüfarzt gibt Ihnen am Ende der Studie aber gern eine Zusammenfassung der Gesamtergebnisse der Studie, wenn Sie das wünschen.

Teil 3:
Datenschutz und Versicherungsschutz

9. Schutz von Daten

Wir schützen Ihre Daten (z.B. Angaben wie Schmerzintensität). Zum Schutz von Daten gibt es in der Schweiz strenge gesetzliche Regelungen.

9.1 Verschlüsselung von Daten

Bei jeder Studie entstehen Daten aus den Untersuchungen (z.B. Angabe der Schmerzintensität). Diese Daten werden dokumentiert. Das passiert meist elektronisch in grossen Tabellen, den sogenannten «Datenerhebungsbögen». Alle Daten werden verschlüsselt dokumentiert. «Verschlüsselt» heisst, dass persönliche Informationen *getrennt* von den Untersuchungsergebnissen aufbewahrt werden. Dazu gibt es eine Liste, die jede Person mit einem eindeutigen Code identifiziert. So stehen z.B. Ihr Name, Ihr Geburtsdatum oder Ihr Wohnort *nicht* direkt im Datenerhebungsbogen. Diese Liste bleibt für die Dauer von min. zehn Jahren am Spital. Nur sehr wenige Fachpersonen werden Ihre unverschlüsselten Daten sehen und zwar nur, um Aufgaben im Rahmen der Studie zu erfüllen. Diese Personen unterliegen der Schweigepflicht.

Am Ende der Studie werden Ihre Daten vollständig anonymisiert, frühstens am Ende der gesetzlich vorgegebenen Aufbewahrungsdauer. Das bedeutet, dass es nicht mehr möglich sein wird, Sie ohne unverhältnismässigen Aufwand zu identifizieren. Zur Anonymisierung werden verschiedene Massnahmen eingesetzt, u.a. die Vernichtung des Codes und der Liste.

Wenn wir Daten weitergeben – an den Sponsor oder an Fachpersonen, die weitere Untersuchungen machen – dann sind die Daten immer verschlüsselt und Ihre persönlichen Daten sind geschützt. Das gilt auch, wenn die Daten ins Ausland weitergegeben werden.

9.2 Sicherer Umgang mit den Daten während der Studie

Der Sponsor ist verantwortlich für den sicheren Umgang mit Ihren Daten aus dieser Studie. Er ist verantwortlich dafür, dass die geltenden Gesetze, z.B. die Datenschutzgesetze, eingehalten werden. Dies gilt auch, wenn (verschlüsselte) Daten für Untersuchungen in Länder verschickt werden, wo die Datenschutzgesetze weniger gut sind. So schützt der Sponsor dieser Studie Ihre Daten: In dieser Studie werden Ihre Daten elektronisch erfasst und übermittelt. Die Daten sind auf einem Server in der Schweiz gespeichert. Trotzdem gibt es immer ein gewisses Restrisiko, dass fremde Personen auf Ihre persönlichen Daten zugreifen (z.B. Risiko von „Hacking“).

9.3 Sicherer Umgang mit Daten nach der Studie

Der Sponsor bleibt auch nach Ende der Studie verantwortlich für den sicheren Umgang mit Ihren Daten und Proben. Das Gesetz schreibt vor, dass alle Studiendokumente, z.B. die Datenerhebungsbögen, für mindestens zehn Jahre aufbewahrt werden.

Nach Abschluss einer Studie werden die Ergebnisse meist in wissenschaftlichen Zeitschriften veröffentlicht. Dazu werden die Ergebnisse durch andere Fachpersonen begutachtet. Ihre verschlüsselten Daten müssen dabei an diese Fachpersonen weitergeleitet werden. Die Daten dürfen allerdings nicht für neue Forschungszwecke weiterverwendet werden. Dafür würde es Ihre separate Einwilligung brauchen (vgl. **Kapitel 9.4**).

9.4 Weiterverwendung Ihrer Daten in anderen, zukünftigen Studien

Ihre Daten aus dieser Studie sind für die zukünftige Forschung sehr wichtig. Daten, die für diese Studie nicht schon vollständig verbraucht wurden, können möglicherweise für andere Studien weiterverwendet werden. Das ist freiwillig. Bitte lesen Sie die zusätzliche Einwilligungserklärung am Schluss des Dokuments genau durch. Unterschreiben Sie bitte die Einwilligung, wenn Sie mit Ihren Daten weitere Forschung in der Zukunft unterstützen möchten. Auch wenn Sie nicht zustimmen, können Sie trotzdem an der Studie teilnehmen.

9.5 Einsichtsrechte bei Kontrollen

Die Durchführung dieser Studie kann überprüft werden. Die Überprüfung geschieht durch Behörden wie die zuständige Ethikkommission. Auch der Sponsor muss solche Überprüfungen machen, damit die Qualität dieser Studie und die Ergebnisse gesichert sind.

Dafür erhalten wenige, speziell dafür ausgebildete Personen Einblick in Ihre persönlichen Daten. Für diese Überprüfung sind die Daten also *nicht* verschlüsselt. Die Personen, die Ihre unverschlüsselten Daten sehen, unterliegen der Schweigepflicht.

10. Versicherungsschutz

Sie sind versichert, wenn Sie durch die Studie – also durch das Schmerzmodell und/oder die offen verabreichten Placebos – einen Schaden erleiden. Das Vorgehen ist gesetzlich geregelt. Dafür hat der Sponsor eine Versicherung abgeschlossen bei Helvetia Versicherungen, Dufourstrasse 40, CH-9001 St. Gallen. Wenn Sie meinen, dass Sie einen Schaden durch die Studie erlitten haben, wenden Sie sich bitte an Ihre Prüfärztin / Ihren Prüfarzt oder den Studienleiter.

Bei Schäden, die auf ein zugelassenes und nach medizinischem Standard angewendetes Arzneimittel / Medizinprodukt zurückzuführen sind oder auch bei Anwendung einer üblichen Therapie aufgetreten wären, gelten dieselben Haftungsregelungen wie bei einer Behandlung ausserhalb einer Studie. In einem solchen Fall übernimmt die Haftpflichtversicherung des Spitals die Kosten / Entschädigung.

Teil 4: Einwilligungserklärungen

Diese Einwilligung besteht aus zwei unabhängigen Einwilligungserklärungen:

- Einwilligungserklärung zur Teilnahme an dieser Studie
- Einwilligungserklärung für die Weiterverwendung von Daten aus dieser Studie in verschlüsselter Form

Bitte lesen Sie dieses Formular sorgfältig durch. Bitte fragen Sie uns, wenn Sie etwas nicht verstehen oder wenn Sie noch etwas wissen möchten. Für die Teilnahme ist Ihre schriftliche Einwilligung notwendig.

Einwilligungserklärung zur Teilnahme an dieser Studie

**BASEC-Nummer**	2023-00296
**Titel der Studie**	Boosting Open-Label Placebo Effects in Acute Induced Pain in Healthy Adults
**Laienverständlicher Titel**	Wirkdauer und Boost-Effekte von offen verabreichten Placebos bei akuten induzierten Schmerzen
**Verantwortliche Institution**<br>(Sponsor mit Adresse)	**Universitätsspital Basel**<br>Klinik Anästhesiologie<br>Schmerztherapie<br>Spitalstrasse 21<br>CH-4031 Basel
**Ort der Durchführung**	Universitätsspital Basel
**Prüfärztin/ Prüfarzt am Studienort**	Dr. med. Tobias Schneider
**Teilnehmerin/ Teilnehmer:**<br>Name und Vorname in Druckbuchstaben:<br>Geburtsdatum:	

- Ich habe mündlich und schriftlich Informationen über die Studie bekommen, und zwar von der Prüfärztin / dem Prüfarzt, die / der unten unterschreibt.
- Die Prüfärztin / der Prüfarzt hat mir den Zweck, den Ablauf und die Risiken der Studie erklärt.
- Ich nehme freiwillig an der Studie teil.
- Ich hatte genügend Zeit, um diese Entscheidung zu treffen. Ich behalte die schriftliche Information und erhalte eine Kopie meiner schriftlichen Einwilligungserklärung.
- Ich kann jederzeit meine Teilnahme beenden. Ich muss nicht erklären, warum. Auch wenn ich die Teilnahme beende, entstehen mir keine Nachteile. Die Daten, die bis dahin gesammelt wurden, bleiben gespeichert und werden im Rahmen der Studie ausgewertet.
- Wenn es besser für meine Gesundheit ist, kann mich die Prüfärztin / der Prüfarzt jederzeit von der Studie ausschliessen.
- Ich habe verstanden, dass meine Daten nur in verschlüsselter Form weitergegeben und/oder ins Ausland gesendet werden. Der Sponsor sorgt dafür, dass der Datenschutz nach Schweizer Standard eingehalten wird.
- Bei Ergebnissen und/oder Zufallsbefunden, die direkt meine Gesundheit betreffen, werde ich informiert. Wenn ich das nicht wünsche, bespreche ich das mit meiner Prüfärztin/ meinem Prüfarzt.
- Die zuständigen Fachpersonen des Sponsors und der Ethikkommission dürfen meine unverschlüsselten Daten zur Kontrolle einsehen. Alle diese Personen unterstehen der Schweigepflicht.
- Ich weiss, dass der Sponsor eine Versicherung abgeschlossen hat. Diese Versicherung bezahlt, wenn ich einen Schaden erleide – aber nur, wenn der Schaden direkt mit der Studie zusammenhängt. Die Haftpflichtversicherung des Spitals versichert mögliche Schäden.
- Ich bin mir bewusst, dass die in der Teilnehmerinformation genannten Pflichten (vgl. **Kapitel 5**) einzuhalten sind.

Ort, Datum, Uhrzeit	Name und Vorname Teilnehmerin / Teilnehmer in Druckbuchstaben<br><br><br>Unterschrift Teilnehmerin / Teilnehmer

Bestätigung der Prüfärztin / des Prüfarztes: Hiermit bestätige ich, dass ich dieser Teilnehmerin / diesem Teilnehmer Art, Bedeutung und Tragweite der Studie erläutert habe. Ich versichere, alle mit dieser Studie stehenden Verpflichtungen nach Schweizer Recht zu erfüllen. Sollte ich im Verlauf der Studie von Aspekten erfahren, welche die Bereitschaft der Teilnehmerin / des Teilnehmers zur Studienteilnahme beeinflussen könnten, werde ich sie / ihn umgehend darüber informieren.

Ort, Datum, Uhrzeit	Name und Vorname der Prüfärztin / des Prüfarztes in Druckbuchstaben<br><br><br>Unterschrift der Prüfärztin / des Prüfarztes

Einwilligungserklärung für Weiterverwendung von Daten in verschlüsselter Form

Diese Einwilligung betrifft Sie nicht im Sinne der persönlichen Teilnahme an einer Studie. «Weiterverwendung» meint, dass Daten über die Zeit Ihrer Studienteilnahme hinaus aufbewahrt und in verschlüsselter Form für weitere Forschung verwendet werden können. Das kann z.B. heissen, dass Daten von Ihnen statistisch ausgewertet werden oder neue Untersuchungen daran gemacht werden.

**BASEC-Nummer:**	2023-00296
**Titel der Studie**	Boosting Open-Label Placebo Effects in Acute Induced Pain in Healthy Adults
**Laienverständlicher Titel**	Wirkdauer und Boost-Effekte von offen verabreichten Placebos bei akuten induzierten Schmerzen
**Teilnehmerin/Teilnehmer:**<br>Name und Vorname in Druckbuchstaben:<br>Geburtsdatum:	

- Ich erlaube, dass meine verschlüsselten Daten aus dieser Studie für die medizinische Forschung weiterverwendet werden dürfen. Sie stehen dann für zukünftige, weitere Forschungsprojekte auf unbestimmte Zeit zur Verfügung.
- Ich habe verstanden, dass die Daten verschlüsselt sind und der Schlüssel sicher aufbewahrt wird.
- Die Daten können im In- und Ausland ausgewertet werden und in einer Datenbank hier oder im Ausland gespeichert werden. Forschungsinstitutionen im Ausland müssen dieselben Standards zum Datenschutz einhalten, wie sie in der Schweiz gelten.
- Ich entscheide freiwillig und kann diesen Entscheid zu jedem Zeitpunkt wieder zurücknehmen. Wenn ich zurücktrete, werden alle meine Daten anonymisiert. Ich informiere lediglich meine Prüfärztin / meinen Prüfarzt und muss diesen Entscheid nicht begründen.
- Normalerweise werden alle Daten zusammengefasst ausgewertet. Wenn sich zufällig ein Ergebnis zeigt, das für meine Gesundheit sehr wichtig ist, werde ich kontaktiert. Wenn ich das nicht wünsche, teile ich dies meiner Prüfärztin / meinem Prüfarzt mit.

Ort, Datum, Uhrzeit	Name und Vorname Teilnehmerin / Teilnehmer in Druckbuchstaben<br><br><br>Unterschrift Teilnehmerin / Teilnehmer

Bestätigung der Prüfärztin / des Prüfarztes: Ich bestätige, dass ich der Teilnehmerin/dem Teilnehmer Art, Bedeutung und Tragweite der Weiterverwendung von Daten erläutert habe.

Ort, Datum, Uhrzeit	Name und Vorname der Prüfärztin / des Prüfarztes in Druckbuchstaben<br><br><br>Unterschrift der Prüfärztin / des Prüfarztes
