# Supplementary material for: Boosting open-label placebo effects in acute induced pain in healthy adults (BOLPAP-study): study protocol of a randomized controlled trial
Source: Front Med (Lausanne). 2024 Feb 14;11:1238878. doi: 10.3389/fmed.2024.1238878 (PMC10900763; doi:10.3389/fmed.2024.1238878)
Supplement: Supplementary file 3 [file Data_Sheet_3.pdf]

# *Supplementary Material*

## 1 Script for Study Intervention (English)

The exact wording of the scripted treatment rationale provided alongside the saline injection, translated from German, will be as following (cf. 2.1 for the German version):

"You have been randomly assigned to get the placebo intervention today. This means that you will receive an injection from us now. These syringes do not contain painkillers, but saline solution. So, it's a placebo. We know from prior research that openly administered placebos are able to reduce pain clearly and in a clinically significant way. This has already been shown in various clinical studies in the last years. This reduction in pain through placebos is triggered, among other things, by the expectation of taking an effective medication. The expectation leads to the release of pain-regulating substances in the brain, which act against pain in the same way as morphine. This does not mean that you have to believe in it, but an open attitude is helpful. For these reasons, we assume that the injection with the placebo can help you to reduce the pain."

In Part 2 of this study, participants will receive another OLP injection, either at a fixed time point (derived from Part 1; if data is inconclusive, at 100 minutes) or on-demand. The injection will be done as described for Part 1, except for the first sentence of the rationale. This sentence will be changed according to the group participants were randomized to. For every subsequent OLP administration, patients will be reminded of the inertness of the injection and that we think that this injection might help with regulating pain.

The exact wording of the reminder of the treatment rationale provided alongside the additional saline injection, translated from German, will be as following (cf. 2.2 for the German version):

"I will now give you another injection of the placebo. As you already know, this injection does not contain a painkiller, but a placebo, which we believe, can help you reduce the pain."

## 2 Script for Study Intervention (German)

Das untenstehende Skript wird direkt vor der Injektion des OLP vorgetragen. Während dem Vortragen des Skripts steht der/die BehandlerIn dem/der PatientIn gegenüber und spricht mit einer ruhigen, freundlichen Stimme. Die Injektion des NaCl 0.9% ist für den/die PatientIn gut sichtbar und die Kochsalzlösung wird langsam über mindestens 10 Sekunden über eine 5 ml-Spritze injiziert.

### 2.1 Teil 1

«Sie wurden nach dem Zufallsprinzip für heute der Placebo-Intervention zugeteilt. Das heisst, dass Sie jetzt eine Injektion von uns erhalten werden.

Diese Spritzen enthalten kein Schmerzmedikament, sondern Kochsalzlösung. Es handelt sich also um ein Placebo. Wir wissen aus der Forschung, dass offen verabreichte Placebos in der Lage sind, Schmerzen deutlich und klinisch bedeutsam zu reduzieren. Dies konnte in den letzten Jahren in verschiedenen klinischen Studien nachgewiesen werden. Diese Schmerzreduktion durch Placebos

wird unter anderem durch die Erwartung, ein wirksames Medikament einzunehmen, ausgelöst. Die Erwartung führt im Gehirn zur Freisetzung von schmerzregulierenden Stoffen, welche auf dieselbe Art und Weise wie Morphium gegen die Schmerzen wirken. Das heisst aber nicht, dass Sie daran glauben müssen, jedoch ist eine offene Haltung hilfreich. Aus diesen Gründen nehmen wir an, dass die Spritze mit dem Placebo Ihnen helfen kann, die Schmerzen zu reduzieren.

Haben Sie noch eine Frage?»

### 2.2 Teil 2

In Teil 2 wird die erste Injektion, abgesehen vom ersten Satz der Erklärung, genau gleich durchgeführt wie in Teil 1. Dieser erste Satz wird entsprechend der Gruppe, in die die TeilnehmerInnen randomisiert wurden, abgeändert.

Vor der zweiten Injektion werden die TeilnehmerInnen an die Erklärung von der ersten Injektion erinnert. Das Skript dafür ist das folgende:

«Ich werde Ihnen nun erneut eine Spritze mit dem Placebo verabreichen. Wie Sie bereits wissen, enthält diese Spritze kein Schmerzmittel, sondern ein Placebo, von dem wir annehmen, dass es Ihnen helfen kann, die Schmerzen zu reduzieren.

Haben Sie noch eine Frage?»

## 3 Script for Control Intervention (English)

The script below will be presented to the participants during the time, in which the study intervention would also be done. The script will be presented analogously to the study intervention. For the German versions of these scripts, cf. 4.

### 3.1 Part 1

"For today, you have been randomly assigned to the control group. This means, you won't get an injection from us today.

The control group is important, because this study is testing a new treatment and we're only able to assess the benefit of this new treatment by comparing intervention and control.

Do you have a question?"

### 3.2 Part 2

With Part 2 we want to assess whether a single repetition of the OLP is beneficial to only one dose. Therefore, the control group will get one OLP injection too, but no repetition. The procedure of the OLP application will be similar to Part 1, except for the first sentence of the rationale. This sentence will be changed according to the group participants were randomized to.

Moreover, participants will be reminded of the importance of the control group. This will be done analogously to Part 1 (cf. 3.1).

## 4 Script for Control Intervention (German)

Das untenstehende Skript wird zum gleichen Zeitpunkt vorgetragen, an dem auch die Studienintervention stattfinden würde. Das Prozedere ist analog zur Studienintervention.

### 4.1 Teil 1

«Sie wurden nach dem Zufallsprinzip für heute der Kontrollgruppe zugeteilt. Das heisst, dass Sie heute keine Injektion von uns erhalten werden.

Die Kontrollgruppe ist wichtig, da diese Studie eine neue Behandlung testet und wir nur im Vergleich von Intervention und Kontrolle den Mehrwert dieser Behandlung beurteilen können.

Haben Sie noch eine Frage?»

### 4.2 Teil 2

In Teil 2 möchten wir beurteilen, ob eine einmalige Repetition des OLP einen Vorteil gegenüber einer einzelnen Dosis bringt. Die Kontrollgruppe erhält deshalb ebenfalls eine OLP-Injektion, aber keine Wiederholung davon. Das Prozedere für die OLP-Applikation wird gleich sein wie in Teil 1, abgesehen vom ersten Satz der Erklärung. Dieser erste Satz wird entsprechend der Gruppe, in die die TeilnehmerInnen randomisiert wurden, abgeändert.

Zudem werden die TeilnehmerInnen wie in Teil 1 an die Wichtigkeit der Kontrollgruppe erinnert (vgl. 4.1).

## 5 Treatment Expectation Questions (German)

Immediately before receiving an OLP injection, participants will answer the following questions:

- «Wie viel Linderung Ihrer Symptome erwarten Sie von der Behandlung?» (0 = keine Linderung / kein Nutzen, 10 = komplette Linderung / sehr grosser Nutzen)
- «Wie viel Nutzen erhoffen Sie sich von der Behandlung?» (0 = keine Linderung / kein Nutzen, 10 = komplette Linderung / sehr grosser Nutzen)

## 6 Intervention Credibility Questions (English)

The participants receiving OLP will answer the following questions after their intervention:

- «How credible did you find the explanation why the placebo treatment can work?" (Not at all, Barely, A little, Fairly, Strong, Extreme)
- "How helpful did you find the explanation why the placebo treatment can work?" (Not at all, Barely, A little, Fairly, Strong, Extreme)
- "During this study, you have received syringes without a drug. On a scale from 0 (not at all) to 10 (extreme), how helpful was the placebo concerning pain regulation?"

## 7 Intervention Credibility Questions (German)

Die TeilnehmerInnen, die eine OLP-Injektion erhalten, werden nach der Intervention folgende Fragen beantworten:

- «Wie glaubwürdig fanden Sie die Erklärung, warum die Placebobehandlung wirken kann?» (überhaupt nicht, minimal, kaum, mässig, stark, sehr stark)
- «Wie hilfreich fanden Sie die Erklärung, warum die Placebobehandlung wirken kann?» (überhaupt nicht, minimal, kaum, mässig, stark, sehr stark)
- «Sie haben im Rahmen dieser Studie Spritzen ohne medizinischen Inhaltsstoff erhalten. Auf einer Skala von 0 (überhaupt nicht hilfreich) bis 10 (extrem hilfreich), wie hilfreich war das Placebo bei der Schmerzregulation?
